# The clinical epidemiology of sickle cell anemia In Africa

**DOI:** 10.1002/ajh.24986

**Published:** 2017-12-18

**Authors:** Alex W. Macharia, George Mochamah, Sophie Uyoga, Carolyne M. Ndila, Gideon Nyutu, Johnstone Makale, Metrine Tendwa, Emily Nyatichi, John Ojal, Mohammed Shebe, Kennedy O. Awuondo, Neema Mturi, Norbert Peshu, Benjamin Tsofa, J. Anthony G. Scott, Kathryn Maitland, Thomas N. Williams

**Affiliations:** ^1^ KEMRI/Wellcome Trust Research Programme, Kilifi Kenya; ^2^ London School of Hygiene and Tropical Medicine London WC1E 7HT United Kingdom; ^3^ INDEPTH Network Accra Ghana; ^4^ Faculty of Medicine Imperial College, St Mary's Hospital London W21NY United Kingdom

## Abstract

Sickle cell anemia (SCA) is the commonest severe monogenic disorders of humans. The disease has been highly characterized in high‐income countries but not in sub‐Saharan Africa where SCA is most prevalent. We conducted a retrospective cohort study of all children 0–13 years admitted from within a defined study area to Kilifi County Hospital in Kenya over a five‐year period. Children were genotyped for SCA retrospectively and incidence rates calculated with reference to population data. Overall, 576 of 18,873 (3.1%) admissions had SCA of whom the majority (399; 69.3%) were previously undiagnosed. The incidence of all‐cause hospital admission was 57.2/100 person years of observation (PYO; 95%CI 52.6–62.1) in children with SCA and 3.7/100 PYO (95%CI 3.7–3.8) in those without SCA (IRR 15.3; 95%CI 14.1–16.6). Rates were higher for the majority of syndromic diagnoses at all ages beyond the neonatal period, being especially high for severe anemia (hemoglobin <50 g/L; IRR 58.8; 95%CI 50.3–68.7), stroke (IRR 486; 95%CI 68.4–3,450), bacteremia (IRR 23.4; 95%CI 17.4–31.4), and for bone (IRR 607; 95%CI 284–1,300), and joint (IRR 80.9; 95%CI 18.1–362) infections. The use of an algorithm based on just five clinical features would have identified approximately half of all SCA cases among hospital‐admitted children with a number needed to test to identify each affected patient of only fourteen. Our study illustrates the clinical epidemiology of SCA in a malaria‐endemic environment without specific interventions. The targeted testing of hospital‐admitted children using the Kilifi Algorithm provides a pragmatic approach to early diagnosis in high‐prevalence countries where newborn screening is unavailable.

## 1 INTRODUCTION

Sickle cell anemia (SCA) is a major global public health concern of which sub‐Saharan Africa bears the greatest load. More than 3 out of 4 of all those affected worldwide are born within the region—almost a quarter of a million new births every year.^1^ The allele responsible, the β^s^ mutation in *HBB*, is the textbook example of a balanced polymorphism in humans.^2^ The sickle mutation has been selected to high population frequencies in many tropical regions because carriers (HbAS) are strongly protected against death from *P. falciparum* malaria^3^; however, homozygotes with SCA suffer chronic ill health and reduced survival^1^ and even today, without specific treatment most children born with SCA in sub‐Saharan Africa will die before their fifth birthday.^4^

Although the clinical epidemiology of SCA has been well described in resource‐rich regions, most notably through the Cooperative Study of Sickle Cell Disease in the United States,^5^ few studies have been conducted in sub‐Saharan Africa.^6^ This is important because the natural history of SCA is likely to differ in the African context for reasons that include the heavy burden of malaria and other infections, more limited access to medical care and high rates of undernutrition.^7^, ^8^

In this study, our aim was to document the common complications of SCA in a typical African hospital. Specifically, we wished to investigate the current and future burdens placed by SCA on African health facilities and the potential utility of targeted screening among specific subgroups of hospital‐admitted children as an alternative to newborn screening in the community.

## 2 METHODS

### 2.1 Study population

The study was conducted on the pediatric wards of Kilifi County Hospital on the Coast of Kenya. This serves as the first referral hospital for Kilifi County where the common causes of admission are similar to those in many hospitals in sub‐Saharan Africa.^9^ In 2000, the Kilifi Health and Demographic Surveillance System was established in a defined area of 891 km^2^ surrounding Kilifi County Hospital, which includes a population of around 100,000 children younger than 14 years.^10^ Approximately 80% of children who are admitted to Kilifi County Hospital reside within the Kilifi Health and Demographic Surveillance System study area.^10^ At the time of sampling, and even to date, there was no routine screening for SCA anywhere in Kenya nor any specific nationally based guidelines regarding management. Hydroxyurea treatment, now commonly used in high‐income countries, was not available.

### 2.2 Study participants

A system of routine clinical surveillance has been operating at Kilifi County Hospital since 1989 through which trained clinicians assess all children at both admission and discharge, and record standard data on a computerized proforma.^10^ A range of laboratory tests are conducted on all admitted children which include a full hemogram, a malaria blood film, and a blood culture.^11^ Admission samples from this surveillance system have been archived at −80°C since 2000. This study included all children younger than 14 years who were residents of the Kilifi Health and Demographic Surveillance System study area and who were admitted to Kilifi County Hospital between January 2000 and December 2004. We chose this study interval because during this period malaria was the commonest cause of pediatric admission^9^ and the incidence of uncomplicated malaria in the surrounding community was between one and three episodes/child/year,^9^ allowing us to address the important question of the relative contribution of malaria to ill health in children with SCA in Africa. Furthermore, this predated the introduction of routine immunization against pneumococcal infections and of the wide‐spread diagnosis and provision of care for children with SCA. As such, the study is broadly representative of the natural history of SCA in the absence of specific interventions. All cases were classified according to their SCA status (SCA ‐ HbSS; non‐SCA ‐ HbAA or HbAS), determined through the genotyping of archived admission samples during 2015 and 2016, and also on the basis of a range of clinical and laboratory features (Table 1). Finally, through careful inspection of their clinical notes, confirmed cases of SCA were further classified according to a number of additional features that were specific to children with SCA but that are not ordinarily captured by our clinical surveillance database. Such complications included pain, hand‐foot syndrome and priapism, as defined in Table 5. Incidence rates were computed through use of the mid‐study population of the Kilifi Health and Demographic Surveillance System study area^10^ stratified by the age‐specific prevalence of SCA. As described in detail previously, we determined the latter by typing for SCA children who we recruited by random sampling throughout the Kilifi Health and Demographic Surveillance System study area into studies undertaken between 1998 and 2005.^12^ Informed consent for inclusion in the hospital surveillance study was obtained from all participants or their parents while permission to test admission samples retrospectively for SCA was provided by the Kenya Medical Research Institute/National Ethical Review Committee. We attempted to trace all SCA‐children identified through this study to offer them counseling and specialist care.

**Table 1:** The clinical phenotypes of hospital‐admitted patients, stratified by SCA status

Syndrome	Non‐SCA *N* = 18,297	SCA *N* = 576	Sensitivity	95% CI	PPV	95% CI
*Clinical syndromes*						
Neonatal conditions	1,839 (10.1)	19 (3.3)	3.3	2.0–5.1	1.0	0.6–1.6
Malaria	5,561 (30.4)	47 (8.2)	8.2	6.1–10.7	0.8	0.6–1.1
Severe malaria	861 (4.7)	11 (1.9)	1.9	1.0–3.4	1.3	0.6–2.3
Severe pneumonia	500 (2.7)	17 (3.0)	3.0	1.7–4.7	3.3	1.9–5.2
Very severe pneumonia	10,836 (59.2)	315 (54.7)	54.7	50.5–58.8	2.8	2.5–3.2
Meningitis/encephalitis	3,076 (16.8)	49 (8.5)	8.5	6.4–12.0	1.6	1.2–2.1
Severe malnutrition	1,595 (8.7)	47 (8.2)	8.2	6.1–10.7	2.9	2.1–3.8
Gastroenteritis	3,417 (18.7)	50 (8.7)	8.7	6.5–11.3	1.4	1.1–1.9
Jaundice	682 (3.7)	107 (18.6)	18.6	15.5–22.0	13.6	11.3–16.2
Cellulitis/pyomyocitis/abcess	333 (1.8)	17 (3.0)	3.0	1.7–4.7	4.9	2.9–7.7
Septic arthritis^a^	12 (0.1)	2 (0.4)	0.4	0–1.3	14.3	1.8–42.8
Osteomyelitis^b^	12 (0.1)	15 (2.6)	2.6	1.5–4.3	55.6	35.3–74.5
Stroke	2 (0.01)	2 (0.4)	0.4	0–1.3	50.0	6.8–93.2
Other	1,687 (9.2)	120 (20.8)	N/A	N/A	N/A	N/A
*Laboratory based syndromes*						
Neonatal bacteremia	160 (0.9)	2 (0.4)	0.4	0–1.3	1.2	0.2–4.4
Bacteremia^c^	956 (5.2)	46 (7.2)	7.2	5.9–10.5	4.6	3.4–6.1
Malaria blood film positive	7,610 (41.6)	98 (17.0)	17.0	14.0–20.3	1.3	1.0–1.6
Severe anemia	1,470 (8.2)	178 (31.3)	31.3	27.5–35.3	10.8	9.3–12.4
*Outcome*						
Blood transfusion	1,623 (8.9)	165 (28.7)	N/A	N/A	N/A	N/A
Death	1,089 (5.9)	32 (5.6)	N/A	N/A	N/A	N/A

Figures in parentheses are column percentages. Clinical phenotypes were defined as follows: “neonatal conditions”—admission to hospital within the first 28 days of life; “malaria”—a fever in the presence of *P. falciparum* parasitemia at any density in children <1 year old or at a density of >2500 parasites/μL in older children; “severe malaria”—malaria in association with the specific complications of prostration, coma, respiratory distress, or a Hb of <50 g/L; “severe” and “very severe pneumonia”—defined as previously described^31^; meningitis/encephalitis—the clinical detection of “neck stiffness,” prostration or coma (defined as a Blantyre Coma Score of <5), or a bulging fontanel; “severe malnutrition”—a mid‐upper‐arm circumference of ≤7.5 cm in children <6 months or of ≤11.5 in children ≥6 months of age; “gastroenteritis”—diarrhoea (3 or more loose watery stools/day) with or without vomiting (3 or more episodes/day); “jaundice”—the clinical recognition of jaundice by the admitting clinician; “osteomyelitis”—bacterial bone infection diagnosed either clinically or with supportive radiological or microbiological evidence; “cellulitis/pyomyositis/abscess”—diagnosed clinically with or without supportive microbiological evidence; “septic arthritis” diagnosed clinically with or without supportive microbiological evidence; and “stroke”—sudden onset of unilateral weakness persisting for more than seven days and for which other diagnoses had been excluded. Some children contribute data to more than one row.

a Blood cultures were positive for nontyphoidal *Salmonella* spp in one SCA case.

b Blood cultures were positive for nontyphoidal *Salmonella* spp in two SCA cases.

c Positive cultures among children with SCD were *Acinetobacter* spp (3; 7%), *Enterobacter cloacae* (1; 2%), *Escherichia coli* (2; 4%), *Haemophilus influenzae* (7; 15%), *Klebsiella pneumoniae* (1; 2%), non‐typhoidal *Salmonella* spp (8; 17%), *Salmonella typhi* (1; 2%), *Staphylococcus aureus* (2; 4%), *Streptococcus pneumoniae* (19; 41%) and other *Streptococcal* spp (2%). Sensitivities and PPVs were computed using the “diagt” command in Stata v14.2.

N/A: not applicable.

### 2.3 Laboratory procedures

Admission hemograms, blood film examinations for malaria, and blood cultures were performed as previously described.^9^, ^11^ We tested cases for SCA by PCR^13^ in 2016, more than 10 years after the period of sample collection, using DNA extracted by proprietary methods [ABI PRISM (Applied BioSystems, CA) or Qiagen DNA Blood Mini Kit (Qiagen, West Sussex, UK)] from archived samples of whole blood collected at the time of their hospital admission. As a consequence, these genotyping results were not available to affected children or to the admitting clinicians during the data collection period. Samples for our denominator estimates—the random population samples described above—were tested by alkaline electrophoresis of fresh blood samples collected into EDTA on cellulose acetate membranes using proprietary methods (Helena, Sunderland, Tyne & Wear, UK).

### 2.4 Statistical analysis

All analyses were conducted using Stata v14.2 (Stata Corp, Timberlake). Continuous data were compared using parametric or nonparametric tests as appropriate, while proportions were compared using the χ^2^ test. Sensitivities and positive predictive values (PPVs) were computed using the “diagt” command. We calculated the incidence of syndrome‐specific admission to Kilifi County Hospital in SCA and non‐SCA children from the mid‐study population of the Kilifi Health and Demographic Surveillance System area and the age‐specific prevalence of SCA among controls.^12^

## 3 RESULTS

A total of 20,574 children <14 years were admitted to the wards of Kilifi County Hospital from within the Kilifi Health and Demographic Surveillance System study area during the study period, of whom 18,873 (92%) were genotyped successfully and included in this analysis (Supporting Information Figure). The demographic, anthropometric and hematological characteristics of children at the point of admission, stratified by SCA status, are summarized in Table 2. We found no evidence for a prior diagnosis in 399 of 576 (69.3%) SCA admissions. Overall, children with SCA accounted for 3.1% of all admissions and were typically older, less‐well nourished, and differed from children without SCA across a range of hematological parameters (Table 2). Furthermore, among those with SCA, previously diagnosed patients were older (50.9 vs. 22.3 months) and better nourished [height‐for‐age *Z* score (HAZ) −1.05 vs. −1.45; *P* = .003] than undiagnosed children (Supporting Information Table S1). Compared with non‐SCA admissions (7610/18292; 42%), those with SCA were less likely to be positive for *P. falciparum* malaria parasites (98/576; 17%; *P* < .0005; Table 1) and parasite densities were significantly lower in those who were infected (Table 2). While mortality among parasite‐positive patients with SCA (4/98; 4.1%) was higher than that in those without SCA (199/7610; 2.6%), this did not reach statistical significance (χ^2^ = 0.81).

**Table 2:** Clinical and laboratory characteristics of hospital‐admitted patients, stratified by SCA status

	Non‐SCA		SCA		
	18,297 (96.9%)		576 (3.1%)		
Characteristic	Mean	95% CI	Mean	95% CI	*P*
Age^a^ (months)	14.0	13.7–14.3	28.8	26.1–31.9	<.0005
WAZ	−1.68	−1.70, −1.66	−1.82	−1.93, −1.71	.056
HAZ	−1.33	−1.35, −1.30	−1.33	−1.45, −1.20	.997
Parasite density (parasites/μL)	27,101	25,644–28,674	3,468	2,238–5,376	<.0005
Hemoglobin (g/L)	92.0	91.6–92.5	65.6	63.1–68.0	<.0005
RCC^a^ (x10^12^/L)	3.75	3.73–3.77	2.33	2.23–2.43	<.0005
MCV (fL)	74.0	73.8–74.2	81.5	80.5–82.5	<.0005
WBC^a^ (×10^9^/L)	12.2	12.1–12.3	22.9	21.7–24.0	<.0005
Platelets^a^ (×10^6^/L)	236	232–239	293	276–310	<.0005

Abbreviations: WAZ, weight‐for‐age Z‐score; HAZ, height‐for‐age Z‐score; RCC, red cell count; MCV, mean cell volume; WBC, white blood count.

*P*‐values in comparison to non‐SCA children were estimated by use of Student's *t*‐tests.

a Geometric mean.

The clinical phenotypes and outcome of hospital admission, together with data on the potential value of such phenotypes as predictors of SCA, are summarized in Table 1. While children with SCA were over‐represented among some clinically defined subgroups, including those with clinically detectable jaundice, severe anemia (Hb < 50 g/L) and a range of specific bacterial infections, they were under‐represented among both children admitted with malaria and those admitted during the neonatal period. Although some syndromes, including jaundice, severe anemia and stroke, were associated with PPVs of >10%, the same diagnoses did not generally provide a sensitive basis for the targeted screening of hospital‐admitted children. We therefore investigated whether a multi‐disease approach to screening might provide a more efficient strategy. To answer this question we developed an algorithm based on a range of clinical syndromes ordered hierarchically on the basis of their sensitivity and PPV profiles. Given that, in general, it is unnecessary to screen children more than once, we limited this analysis to index admissions for SCA‐patients admitted more than once. We re‐classified each syndrome in this hierarchy to be mutually exclusive of the preceding syndrome, allowing us to estimate the cumulative PPVs and numbers needed to test (NNT) to identify each affected case (Table 3). From this analysis we conclude that by testing all children presenting with just one of five syndromes—stroke, severe anemia, jaundice, septic arthritis and cellulitis, pyomyositis, or abscess we would have identified 47% of all those with SCA by testing only 14% of all those admitted: a NNT to identify each affected child of 14. Testing all admitted children would have been more sensitive (100%) but at the expense of a NNT of 47.

**Table 3:** The potential utility of targeted approaches to SCA screening among patients admitted to the pediatric ward at Kilifi County Hospital

Syndrome	Non‐SCA *N* = 18,297	SCA *N* = 399	Cumulative Sensitivity (%)	PPV (%)	NNT	Cumulative NNT	Proportion of all admissions (%)
Stroke	2 (0.01)	1 (0.25)	0.25	33.3	3	3	0.02
Severe anemia	1,470 (8.03)	130 (32.6)	32.8	8.1	12	12	8.6
Jaundice	634 (3.47)	38 (9.52)	42.4	5.7	18	13	12.2
Septic arthritis	12 (0.07)	2 (0.50)	42.9	14.3	7	13	12.2
Cellulitis/pyomyositis/abcess	323 (1.77)	14 (3.51)	46.4	4.2	24	14	14.0
Bacteremia	780 (4.26)	21 (5.26)	51.6	2.6	38	17	18.3
Osteomyelitis	8 (0.04)	2 (0.50)	52.1	20.0	5	17	18.4
Very severe pneumonia	8,999 (49.2)	114 (28.6)	80.7	1.3	80	39	67.1
Severe pneumonia	397 (2.17)	7 (1.75)	82.5	1.7	58	39	69.3
Severe malnutrition	440 (2.40)	7 (1.75)	84.2	1.6	64	40	71.7
Other	5232 (28.6)	63 (15.8)	100.0	1.2	84	47	100.0

Abbreviations: PPV, positive predictive value; NNT, number needed to test.

Values were computed from the data summarized in Table 1 using the “diagt” command in Stata v14.2. Syndromes were defined as described in Table 1.

The incidence rate ratios (IRRs) for admission with various syndromes in SCA compared to non‐SCA children are shown in Table 4 while the equivalent incidence rates are summarized in Supporting Information Table S2. The overall incidence rates for all‐cause admission were 57.2/100 person years of observation (PYO) (95% CI 52.6–62.1) and 3.7/100 PYO (95% CI 3.7–3.8) in SCA and non‐SCA children respectively (IRR 15.3; 95% CI 14.1–16.6). However, in contrast to non‐SCA children, the incidence rates for admission with a range of syndromes rose strongly with age among those with SCA and, as a result, we present these data stratified by age category (Table 4 and Supporting Information Table S2). Incidence rates were significantly higher among SCA children for the majority of diagnoses at all ages beyond the neonatal period, but were especially high for stroke (IRR 486; 95% CI 68.4–3450), bacteremia (IRR 23.4; 95% CI 17.4–31.4) and for bone (IRR 607; 95% CI 284–1300) and joint (IRR 80.9; 95% CI 18.1–362) infections (Table 4). The raised IRRs for many diagnoses were most marked among undiagnosed children (Supporting Information Table S3). The most common bacteremic infections were due to non‐typhi *Salmonella* species, *Haemophilus influenzae* and *Streptococcus pneumoniae* (Table 1). Severe anemia complicated 31.3% of all SCA admissions while 28.7% received a blood transfusion. This was reflected in an overall IRR for severe anemia of 58.8 (95% CI 50.3–68.7)/100 PYO, and rose with age from 6.7 (95% CI 4.6–9.7)/100 PYO in the age group 0–11 months to 165 (95% CI 135–201)/100 PYO in children over 2 years of age. Although malaria parasitemia was more common among SCA children with than without anemia (24.2% vs. 14.1%, respectively; *P* = .003), blood films were negative in the majority of anemic cases. Finally, the incidence rates for a range of specific complications of SCA are summarized in Table 5. While the incidence of admission with pain was 12.7 episodes/100 PYO overall, this was strongly age dependent and was highest (27.2 episodes/100 PYO) among children more than 2 years old. No episodes of priapism were reported during the period of study.

**Table 4:** IRRs for admission to hospital with specific clinical diagnoses in SCA versus non‐SCA children

	0–13 Years		0–11 Months		12–23 Months		2–13 Years	
Diagnosis	IRR	95% CI	IRR	95% CI	IRR	95% CI	IRR	95% CI
*Clinical syndromes*								
All cause hospital admission	15.3	14.1–16.6	1.7	1.4–2.0	6.8	5.5–8.2	45.4	40.8–50.5
Neonatal conditions	0.7	0.4–1.1	0.7	0.4–1.1	N/A	N/A	N/A	N/A
Malaria	4.10	3.1–5.5	0.8	0.5–1.4	1.4	0.7–2.9	9.4	6.5–13.6
Severe malaria	6.2	3.4–11.2	3.1	1.3–7.6	5.1	1.9–13.6	3.8	0.9–15.2
Severe pneumonia	16.5	10.2–26.8	2.5	1.3–5.1	17.8	7.8–40.6	28.2	9.0–89.0
Very severe pneumonia	14.1	12.6–15.8	1.8	1.4–2.2	6.3	4.8–8.2	42.6	36.6–49.5
Meningitis/encephalitis	7.7	5.8–10.3	1.3	0.9–1.8	7.2	3.4–15.3	18.8	11.3–31.4
Severe malnutrition	14.3	10.7–19.1	2.20	1.4–3.5	9.5	5.8–15.6	35.8	20.6–62.4
Gastroenteritis	7.1	5.4–9.4	1.6	1.1–2.4	3.3	1.9–5.8	12.7	6.8–23.7
Jaundice	76.2	62.1–93.4	2.2	1.2–4.0	116	59.4–228	467.4	359–608
Cellulitis/pyomyositis/abcess	24.8	15.2–40.4	3.5	1.3–9.4	14.6	5.4–39.9	57.0	29.1–111
Septic arthritis	80.9	18.1–362	32.2	3.4–310.1	N/A	N/A	101.4	12.9–800
Osteomyelitis	607	284–1300	96.8	6.1–1550	844	87.8–8120	1003	426–2360
Stroke	486	68–3450	N/A	N/A	N/A	N/A	456	41.4–5032
Other	35	29–42	2.0	0.8–4.7	9.7	5.6–16.8	87.7	71.60–107.50
*Laboratory features and outcomes*								
Neonatal bacteremia	0.8	0.2–3.3	0.8	0.2–3.3	N/A	N/A	N/A	N/A
Bacteremia	23.4	17.4–31.4	3.4	2.0–5.5	12.5	6.2–25.4	63.7	41.4–98.2
Malaria blood film positive	6.3	5.1–7.6	1.0	0.7–1.7	2.8	1.8–4.4	13.8	10.7–17.7
Severe anemia	58.8	50.3–68.7	6.7	4.6–9.7	22.5	15.7–32.3	165	135–201
Transfused	49.4	42.1–57.9	3.8	2.5–5.7	20.3	13.8–29.7	165	135–201
Died	14.3	10.0–20.3	2.0	1.2–3.5	12.1	5.3–27.3	37.3	21.4–65.0

The IRRs for each syndrome and age group were estimated using a series of Poisson regression models. In each syndrome‐specific model the count of that syndrome was the outcome variable and the indicator for SCA was the explanatory variable. The exposure time in each model was PYO estimated as described in the methods. The mid‐study population of the Kilifi Health and Demographic Surveillance System study area was 615 for children 0–28 days, 8,173 for children aged 0–11 months, 7,964 for those aged 12–23 months, and 81,857 for 2–13 year olds; the proportions with SCA in these age groups were 1.52% (95% CI 0.19–2.85), 1.02% CI 0.44–2.01), 0.35% (0.35–1.96), and 0.11% (0.03–0.28), respectively. In each age group, the number of PYO was calculated from the product of the mid‐study population, the prevalence of SCA and the duration of the study in years. The figures for each group were as follows non‐SCA: 0–28 days 36,341; 0–11 months 40,447; 12–23 months 39,679; 2–13 years 408,837; 0–13 years 488,963; SCA 0–28 days 559; 0–11 months 418; 12–23 months 141; 3–13 years 448; 0–13 years 1007.

N/A, not applicable.

**Table 5:** The incidence of hospital admission with a range of SCA‐specific complications for which data were not routinely captured through our ward surveillance system

	0–13 years		0–11 months		12–23 months		2–13 years	
Complication	*n*	Incidence (95% CI)	*n*	Incidence (95% CI)	*n*	Incidence (95% CI)	*n*	Incidence (95% CI)
Pain	128	12.7 (10.6,15.1)	0	–	6	4.3 (1.6,9.3)	122	27.2 (22.6,32.5)
Hand‐foot syndrome	15	1.5 (0.8,2.4)	4	1.0 (0.3,2.5)	4	2.8 (0.8,7.3)	7	1.6 (0.6,3.2)
Arthralgia	34	3.4 (2.3,4.7)	0	–	1	0.7 (0,4.0)	33	7.4 (5.1,10.3)

PYO were derived as described in Table 4. Specific features were determined through physical inspection of the clinical notes of all children with SCA. Pain, any mention of un‐explained pain; hand‐foot syndrome, painful swelling of hands or feet; arthralgia, specific mention of pain in any joint. Some patients manifest more than one complication. No episodes of priapism were identified during the course of this study. Some patients manifest more than one complication.

## 4 DISCUSSION

An estimated 312,000 babies are born with SCA worldwide every year.^7^ Approximately 75% of these births occur in sub‐Saharan Africa^7^ where historically, the disease has been widely neglected.^14^ As a consequence, 50–90% of those affected die, usually undiagnosed, during early childhood.^4^ These figures are reflected in estimates from the Word Health Organization (WHO) which suggest that in many parts of the continent SCA may account for between 5 and 16% of all deaths among children <5 years old.^15^ Despite these figures, few reports have described the epidemiology of SCA within the region and those studies that have been conducted have largely focused on symptomatic children whose disease may well have been modified by supportive interventions.^16^

Our study used a retrospective design in which we exploited data and samples collected through routine surveillance at Kilifi County Hospital to study the epidemiology of SCA in an African population under high malaria exposure. To a large extent, this retrospective approach overcomes the potential biases that may be associated with a known diagnosis or by the widespread provision of supportive interventions.

The overall incidence of admission to hospital among children with SCA 0–13 years was 57.2 (95% CI 52.6–62.1)/100 PYO. However, this was strongly age‐dependent and rose from 29.7 (95% CI 24.7–35.4)/100 PYO in children 0–11 months to 78.6 (95% CI 70.6–87.2)/100 PYO in the age‐group 3–13 years. For a number of reasons, we suggest that these figures reflect a significant underestimate of the total impact of SCA within our study population. First, while Kilifi County Hospital is the main provider of medical care within the study area, it is likely that some children will have sought care in hospitals beyond the study borders. Second, it is likely that the costs associated with medical consultation and hospital admission will have deterred some parents from seeking care. As a result, our study may not have captured all significant events among resident children with SCA.

In contrast to observations made in resource‐rich countries through studies like the US cooperative study for sickle cell disease,^5^ where hand‐foot syndrome was a common presenting feature of SCA, its incidence was low in the current study. While this might simply reflect methodological issues, it could also result from a true difference in the clinical phenotype of SCA within our study population, potentially explained by genetic or environmental factors. Conversely, severe anemia was significantly more common in our study than in reports from the north. For example, while the overall incidence of anemia in the cooperative study was <5/100 PYO,^5^ in our study it was 17.7/100 PYO and reached 26.1/100 PYO among children >2 years old. More than 30% of SCA admissions were severely anemic and 28.7% received one or more transfusions—almost one out of every 10 pediatric transfusions administered at Kilifi County Hospital during the study period. While the design of our study does not allow us to determine the precise etiology of these anemic episodes, the most likely explanations for this geographic discrepancy relate to malaria or nutrition. Although children with SCA are thought to be partially resistant to malaria infection,^17^ a theory supported by the lower prevalence and density of *P. falciparum* infections seen in this study, the disease can take a particularly fulminant course when patients are infected.^18^ While direct evidence of malaria infection was only seen in a quarter of these children, this does not exclude a wider role for malaria through, for example, previous, recurrent or pre‐treated infections. Regardless of the cause, it is clear that in sub‐Saharan Africa, severe anemia places a heavy burden both on children with SCA, their families and on medical services constrained by limited resources. A particular concern relates to blood safety. Even in well‐resourced areas, transfusions are associated with risks and are not administered without careful consideration.^19^ Specific factors like the frequent use of non‐leuko‐depleted whole blood, limited cross‐matching procedures and suboptimal storage conditions magnify these risks in sub‐Saharan Africa,^20^ and it is for such reasons that the WHO recommends restricting transfusions to the children in greatest need.^20^ For all these reasons, early life screening could have a marked impact on anemia through the targeted prevention of malaria and by helping clinicians to make more informed decisions about when and how to transfuse affected children.^21^

In common with other regions,^5^, ^22^, ^23^, ^24^ and as described in detail previously,^12^ bacterial infections were an important reason for hospital admission among children with SCA in our study. Similarly, despite design differences, both our rate and age‐pattern of painful crises were broadly similar to those seen in the Cooperative Study.^5^ Conversely, the incidence of stroke, a major complication of SCA in resource‐rich regions,^1^, ^5^ was significantly lower, most likely reflecting the low rates of survive to the age at which the incidence of stroke begins to rise. Of particular note, the raised incidence of many complications, including malaria, anemia, bacterial infections, and in‐hospital death, was most pronounced among undiagnosed children. This may reflect improved parental education concerning SCA and adherence to antimalarial and antibacterial prophylaxis by children once diagnosed.

While newborn screening would be the best way to identify children with SCA and direct them towards treatment as early in life as possible, this is challenging in sub‐Saharan Africa^14^ and there are few locations within the region where such programs have been established successfully.^25^, ^26^, ^27^ A major rationale for conducting this study was therefore to investigate the potential utility of screening among specific sub‐groups of hospital‐admitted children as a pragmatic alternative. This approach is widely practiced for HIV where, for example, the WHO recommend provider‐initiated testing and counselling for all children presenting to health facilities in low‐income countries.^28^ Although individually none of the clinical syndromes investigated were sufficiently specific to make them especially useful, a number of factors were associated with PPVs of >10%. These included clinical jaundice, severe anemia, bone and joint infections and stroke. We have shown that through the use of a clinical algorithm based on just 5 diagnoses, we would have identified almost half of all SCA‐children within the patient population with a NNT of only 14. It is likely that more children could have been identified with a lower NNT had we included other characteristic complications such as hand‐foot syndrome and unexplained pain in our algorithm. Although our study design meant that we lacked the control data for such syndromes that would have enabled us to test this definitively, in practice, we suggest that by including in our algorithm children admitted with such characteristic features of SCA would improve its performance at limited additional cost. While blood film microscopy or rapid methods such as the sodium metabisulphite sickling test represent potential approaches to screening, they both require a basic laboratory staffed by a trained technician and lack both specificity and sensitivity with regard to differentiating trait from disease. However, the recent development of simple and accurate point‐of‐care testing devices for SCA^29^ now makes opportunistic testing at the bed‐side much more practical. We recommend that where newborn screening is not available, hospitals in Africa should use the Kilifi Algorithm coupled to point‐of‐care testing as a pragmatic alternative.

In summary, we have described the clinical epidemiology of SCA within a malaria‐exposed population in Africa. We highlight important differences in comparison to descriptions from resource‐rich regions including a significantly higher incidence of severe anemia. We show that targeted screening of hospital‐admitted children based on a small number of easily recognized syndromes provides a practical and efficient way to identify a high proportion of affected children and introduce them to care. If the world continues to ignore the problem of SCA, the disease will continue to result in high child‐mortality and threaten the ability of many countries in sub‐Saharan Africa to meet their Sustainable Development Goals.^30^ The need is urgent for a renewed and increased focus on SCA by both African governments and the international community.

## DECLARATION OF INTERESTS

The authors declare no competing interests.

## AUTHOR CONTRIBUTIONS

All authors read and approved the final version of the manuscript.

*Designed the study*: Macharia, Uyoga, Williams

*Conducted the literature review*: Macharia, Uyoga, Williams

*Sample preparation and analysis*: Macharia

*Analyzed data*: Williams, Ndila, Nyutu, Mochamah, Nyutu

*Interpretation of the data*: Macharia, Mochamah, Uyoga, Ndila, Nyutu, Makale, Tendwa, Nyatichi, Ojal, Shebe, Awuondo, Mturi, Peshu, Tsofa, Scott, Maitland, Williams

*Wrote the first draft of the article*: Williams, Macharia.

## Supporting information

Additional Supporting Information may be found online in the supporting information tab for this article.

Supporting Information
