# Supplementary material for: The clinical epidemiology of sickle cell anemia In Africa
Source: Am J Hematol. 2017 Dec 18;93(3):363–70. doi: 10.1002/ajh.24986 (PMC6175377; doi:10.1002/ajh.24986)
Supplement: Supplementary file 1 — Supporting Information [file AJH-93-363-s001.docx]

**Headings and footnotes for supplementary Tables and Figure**

**Supplementary Table 1**

**Heading:**

Clinical and laboratory characteristics of hospital-admitted SCA patients, stratified by pre-existing diagnosis.

**Footnote:**

p-values in comparison to previously diagnosed-SCA were estimated by Student’s *t*-test. ^$^Geometric mean.

**Supplementary Table 2**

**Heading:**

Incidence of admission to hospital with a range of clinical conditions in SCA and non-SCA children.

**Footnote:**

Incidence rates were derived from the SCA-specific event rates and the person years of exposure for SCA and non-SCA patients derived as described in the methods as described in Table 4. Some patients manifest more than one complication and are therefore included in more than one row.

**Supplementary Table 3**

**Heading:**

The incidence rates and incidence rate ratios for hospital admission among children with SCA, stratified by diagnostic status.

**Footnote:**

Some children contribute data to more than one row. Definitions can be found in the text. Figures in parentheses show 95% CIs

**Supplementary Figure**

**Heading:**

Patients included in the study of pediatric admissions.

**Supplementary Table 1.**

| **Characteristic** | **Diagnosed-SCA**  **177 (30.7%)** | | **Undiagnosed-SCA**  **399 (69.3%)** | | ***P*** |
| --- | --- | --- | --- | --- | --- |
|  | **Mean** | **95% CI** | **Mean** | **95% CI** |  |
| Age^$^ (months) | 50.9 | 45.6-56.9 | 22.3 | 19.6-25.4 | <0.0005 |
| WAZ | -1.63 | -1.79-1.46 | -1.91 | -2.05-1.77 | 0.02 |
| HAZ | -1.05 | -1.25-0.85 | -1.45 | -1.60-1.30 | 0.003 |
| Parasite density^$^ (parasites/μl) | 2,392 | 1,122-5,101 | 4,087 | 2,373-7,041 | 0.27 |
| Hemoglobin (g/L) | 64.3 | 61.1-67.6 | 66.1 | 62.8-69.4 | 0.52 |
| RCC^$^ (x10^12^/L) | 2.31 | 2.15-2.48 | 2.34 | 2.22-2.47 | 0.78 |
| MCV (fL) | 82.5 | 80.9-84.1 | 81.1 | 79.8-82.4 | 0.22 |
| WBC^$^ (x10^9^/L) | 25.5 | 23.7-27.5 | 21.8 | 20.4-23.2 | 0.003 |
| Platelets^$^ (x10⁶/L) | 311 | 283-342 | 284 | 265-305 | 0.14 |

**Supplementary Table 2**

| **Diagnosis** | **0-13 years** | | | | **0-11 months** | | | | **12-23 months** | | | | **3-13 years** | | | |
| --- | --- | --- | --- | --- | --- | --- | --- | --- | --- | --- | --- | --- | --- | --- | --- | --- |
|  | **non-SCA** | | **SCA** | | **non-SCA** | | **SCA** | | **non-SCA** | | **SCA** | | **non-SCA** | | **SCA** | |
|  | **N** | **Incidence**  **(95% CI)** | **n** | **Incidence**  **(95% CI)** | **n** | **Incidence**  **(95% CI)** | **n** | **Incidence**  **(95% CI)** | **n** | **Incidence**  **(95% CI)** | **n** | **Incidence**  **(95% CI)** | **n** | **Incidence**  **(95% CI)** | **n** | **Incidence**  **(95% CI)** |
| ***Clinical syndromes*** |  |  |  |  |  |  |  |  |  |  |  |  |  |  |  |  |
| All cause hospital admission | 18297 | 3.7  (3.7-3.8) | 576 | 57.2  (52.6-62.1) | 7056 | 17.5  (17.0-17.9) | 124 | 29.7  (24.7-35.4) | 4164 | 10.5  (10.2-10.8) | 100 | 70.9  (57.7-86.3) | 7077 | 1.7  (1.7-1.8) | 352 | 78.6  (70.6-87.2) |
| Neonatal conditions^$^ | 1839 | 5.1  (4.8-5.3) | 19 | 3.4  (2.1-5.3) | 1839 | 5.1  (4.8-5.3) | 19 | 3.4  (2.1-5.3) | N/A | N/A | N/A | N/A | N/A | N/A | N/A | N/A |
| Malaria | 5561 | 1.1  (1.1-1.2) | 47 | 4.7  (3.4-6.2) | 1423 | 3.5  (3.3-3.7) | 12 | 2.9  (1.5-5.0) | 1409 | 3.6  (3.4-3.7) | 7 | 5.0  (2.0-10.2) | 2729 | 0.7  (0.6-0.7) | 28 | 6.3  (4.2-9.0) |
| Severe malaria | 861 | 0.2  (0.2-0.2) | 11 | 1.1  (0.6-2.0) | 156 | 0.4  (0.3-0.5) | 5 | 1.2  (0.4-2.8) | 222 | 0.6  (0.5-0.6) | 4 | 2.8  (0.8-7.3) | 483 | 0.1  (0.1-0.1) | 2 | 0.5  (0.1-1.6) |
| Severe pneumonia | 500 | 0.1  (0.1-0.1) | 17 | 1.7  (1.0-2.7) | 308 | 0.8  (0.7-0.9) | 8 | 1.9  (0.8-3.8) | 95 | 0.2  (0.2-0.3) | 6 | 4.3  (1.6-9.3) | 97 | 0.024  (0.019-0.029) | 3 | 0.7  (0.1-2.0) |
| Very severe pneumonia | 10836 | 2.2  (2.2-2.3) | 315 | 31.3  (27.9-34.9) | 4485 | 11.1  (10.8-11.4) | 81 | 19.4  (15.4-24.1) | 2559 | 6.5  (6.2-6.7) | 57 | 40.4  (30.6-52.4) | 3792 | 0.9  (0.9-1.0) | 177 | 39.5  (33.9-45.8) |
| Meningitis/encephalitis | 3076 | 0.6  (0.6-0.7) | 49 | 4.9  (3.6-6.4) | 2076 | 5.1  (4.9-5.4) | 27 | 6.5  (4.3-9.4) | 272 | 0.7  (0.6-0.8) | 7 | 5.0  (2.0-10.2) | 728 | 0.2  (0.2-0.2) | 15 | 3.4  (1.9-5.5) |
| Severe malnutrition | 1595 | 0.3  (0.3-0.3) | 47 | 4.7  (3.4-6.2) | 790 | 2.0  (1.8-2.1) | 18 | 4.3  (2.6-6.8) | 474 | 1.2  (1.1-1.3) | 16 | 11.4  (6.5-18.4) | 331 | 0.08  (0.073-0.090) | 13 | 2.9  (1.6-5.0) |
| Gastroenteritis | 3417 | 0.7  (0.7-0.7) | 50 | 5.0  (3.7-6.6) | 1605 | 4.0  (3.8-4.2) | 27 | 6.5  (4.3-9.4) | 1094 | 2.8  (2.6-2.9) | 13 | 9.2  (4.9-15.8) | 718 | 0.2  (0.2-0.2) | 10 | 2.2  (1.1-4.1) |
| Jaundice | 682 | 0.1  (0.1-0.2) | 107 | 10.6  (8.7-12.8) | 489 | 1.2  (1.1-1.3) | 11 | 2.6  (1.3-4.7) | 29 | 0.07  (0.05-0.11) | 12 | 8.5  (4.4-14.9) | 164 | 0.04  (0.034-0.047) | 84 | 18.8  (15.0-23.2) |
| Cellulitis/pyomyositis/abcess | 333 | 0.1  (0.1-0.1) | 17 | 1.7  (1.0-2.7) | 112 | 0.3  (0.2-0.3) | 4 | 1.0  (0.3-2.5) | 77 | 0.2  (0.2-0.2) | 4 | 2.8  (0.8-7.3) | 144 | 0.035  (0.030-0.041) | 9 | 2.0  (0.9-3.8) |
| Septic arthritis | 12 | 2.5x10^-3^  (5.0x10^-5^-1.5x10^-3^) | 2 | 0.2  (0-0.7) | 3 | 7.4x10^-3^  (1.5x10^-3^-2.2x10^-2^) | 1 | 0.2  (0-1.3) | 0 | - | 0 | - | 9 | 2.2x10^-3^  (1.0X10^-3^-4.2x10^-3^) | 1 | 0.2  (0.01-1.24) |
| Osteomyelitis | 12 | 2.5x10^-3^  (5.0x10^-5^-1.5x10^-3^) | 15 | 1.5  (0.8-2.5) | 1 | 2.5x10^-3^  (6.3x10^-5^-1.4x10^-2^) | 1 | 0.2  (0-1.3) | 1 | 2.5x10^-3^  (6.4x10^-5^-1.4x10^-2^) | 3 | 2.1  (0.4-6.2) | 10 | 2.5x10^-3^  (1.2x10^-3^-4.5x10^-3^) | 11 | 2.5  (1.2-4.4) |
| Stroke | 2 | 4.1x10^-4^  (5.0x10^-5^-1.5x10^-3^) | 2 | 0.2  (0-0.7) | 0 | - | 0 | - | 0 | - | 1 | 0.7  (0-4.0) | 2 | 4.9x10^-4^  (5.9x10^-5^-1.8x10^-3^) | 1 | 0.2  (0.01-1.24) |
| Other | 1687 | 0.4  (0.3-0.4) | 120 | 11.9  (9.9-14.3) | 248 | 0.6  (0.5-0.7) | 5 | 1.2  (0.4-2.8) | 378 | 1.0  (0.9-1.1) | 13 | 9.2  (4.9-15.8) | 1061 | 0.3  (0.2-0.3) | 102 | 22.8  (18.6-27.6) |
| ***Laboratory features and outcomes*** |  |  |  |  |  |  |  |  |  |  |  |  |  |  |  |  |
| Neonatal bacteremia^$^ | 160 | 0.4  (0.4-0.5) | 2 | 0.4  (0-1.3) | 160 | 0.4  (0.4-0.5) | 2 | 0.4  (0-1.3) | N/A | N/A | N/A | N/A | N/A | N/A | N/A | N/A |
| Bacteremia | 956 | 0.2  (0.2-0.2) | 46 | 4.6  (3.3-6.1) | 461 | 1.1  (1.0-1.3) | 16 | 3.8  (2.2-6.2) | 180 | 0.5  (0.4-0.5) | 8 | 5.7  (2.5-11.2) | 315 | 0.08  (0.07-0.09) | 22 | 4.9  (3.1-7.4) |
| Malaria blood film positive | 7610 | 1.6  (1.5-1.6) | 98 | 9.7  (7.9-11.9) | 1579 | 3.9  (3.7-4.1) | 17 | 4.1  (2.4-6.5) | 1990 | 5.0  (4.8-5.2) | 20 | 14.2  (8.7-22.0) | 4041 | 1.0  (1.0-1.0) | 61 | 13.6  (10.4-17.5) |
| Severe anemia | 1470 | 0.3  (0.3-0.3) | 178 | 17.7  (15.2-20.5) | 422 | 1.0  (1.0-1.2) | 29 | 6.9  (4.7-10.0) | 400 | 1.0  (0.9-1.1) | 32 | 22.7  (15.5-32.0) | 648 | 0.2  (0.2-0.2) | 117 | 26.1  (21.6-31.3) |
| Transfused | 1623 | 0.3  (0.3-0.3) | 165 | 16.4  (14.0-19.1) | 610 | 1.5  (1.4-1.6) | 24 | 5.7  (3.7-8.5) | 389 | 1.0  (0.9-1.1) | 28 | 19.9  (13.2-28.7) | 624 | 0.2  (0.1-0.2) | 113 | 25.2  (20.8-30.3) |
| Died | 1089 | 0.2  (0.2-0.2) | 32 | 3.2  (2.2-4.5) | 631 | 1.56  (1.44-1.69) | 13 | 3.1  (1.7-5.3) | 140 | 0.4  (0.3-0.4) | 6 | 4.3  (1.6-9.3) | 318 | 0.08  (0.07-0.09) | 13 | 2.9  (1.6-5.0) |

**Supplementary Table 3**

| **Diagnosis** | **Non-SCA** | **Incidence**  **(95% CI)** | **Un-diagnosed**  **SCA** | **Incidence**  **(95% CI)** | **IRR** | **Diagnosed**  **SCA** | **Incidence**  **(95% CI)** | **IRR** |
| --- | --- | --- | --- | --- | --- | --- | --- | --- |
| ***Clinical syndromes*** |  |  |  |  |  |  |  |  |
| All cause hospital admission | 18297 | 3.74  (3.69-3.80) | 399 | 39.6  (35.8-43.7) | 10.6  (9.6-11.7) | 177 | 17.6  (15.1-20.4) | 4.7  (4.1-5.4) |
| Neonatal conditions^$^ | 1839 | 5.06  (4.83-5.30) | 19 | 3.4  (2.05-5.31) | 0.7  (0.4-1.1) | 0 | 0  (0-0.66) | 0 |
| Malaria | 5561 | 1.14  (1.11-1.17) | 33 | 3.28  (2.26-4.6) | 2.9  (2.0-4.1) | 14 | 1.39  (0.76-2.33) | 1.2  (0.7-2.1) |
| Severe malaria | 861 | 0.18  (0.16-0.19) | 10 | 0.99  (0.48-1.83) | 5.6  (3.0-10.5) | 1 | 0.1  (0.003-0.55) | 0.6  (0.1-4.0) |
| Severe pneumonia | 500 | 0.1  (0.09-0.11) | 13 | 1.29  (0.69-2.2) | 12.6  (7.3-21.9) | 4 | 0.4  (0.11-1.02) | 3.9  (1.5-10.4) |
| Very severe pneumonia | 10836 | 2.22  (2.17-2.26) | 236 | 23.4  (20.5-26.6) | 10.6  (9.3-12.0) | 79 | 7.8  (6.21-9.78) | 3.5  (2.8-4.4) |
| Meningitis/encephalitis | 3076 | 0.63  (0.61-0.65) | 44 | 4.37  (3.17-5.87) | 6.9  (5.2-9.5) | 5 | 0.5  (0.16-1.16) | 0.8  (0.3-1.9) |
| Severe malnutrition | 1595 | 0.33  (0.31-0.34) | 44 | 4.37  (3.17-5.87) | 13.4  (9.9-18.1) | 3 | 0.3  (0.06-0.87) | 0.9  (0.3-2.8) |
| Gastroenteritis | 3417 | 0.7  (0.68-0.72) | 43 | 4.27  (3.09-5.75) | 6.1  (4.5-8.3) | 7 | 0.7  (0.28-1.43) | 1.0  (0.5-2.1) |
| Jaundice | 682 | 0.14  (0.13-0.15) | 59 | 5.86  (4.46-7.56) | 42  (32.2-54.8) | 48 | 4.8  (3.51-6.32) | 34.2  (25.5-45.8) |
| Cellulitis/pyomyositis/abcess | 333 | 0.07  (0.06-0.08) | 14 | 1.39  (0.76-2.33) | 20.4  (12.0-34.8) | 3 | 0.3  (0.06-0.87) | 4.4  (1.4-13.6) |
| Septic arthritis | 12 | 2.45x10^-4^  (4.95x10^-5^-1.48x10^-3^) | 2 | 0.2  (0.02-0.72) | 80.9  (18.1-362) | 0 | 0  (0-0.37) | 0 |
| Osteomyelitis | 12 | 2.45x10^-4^  (4.95x10^-5^-1.48x10^-3^) | 8 | 0.79  (0.34-1.57) | 324  (132-792) | 7 | 0.7  (0.28-1.43) | 283  (112-719) |
| Stroke | 2 | 4.09x10^-4^  (4.95x10^-5^-1.48x10^-3^) | 1 | 0.1  (0.003-0.55) | 243  (22-2677) | 1 | 0.1  (0.003-0.55) | 243  (22-2677) |
| Other | 1687 | 0.35  (0.33-0.36) | 60 | 5.96  (4.55-7.67) | 17.3  (13.3-22.3) | 60 | 6.0  (4.55-7.67) | 17.3  (13.3-22.3) |
| ***Laboratory features and outcomes*** |  |  |  |  |  |  |  |  |
| Neonatal bacteremia^$^ | 160 | 0.44  (0.37-0.51) | 2 | 0.36  (0.04-1.29) | 0.8  (0.2-3.3) | 0 | 0  (0-0.66) | 0 |
| Bacteremia | 956 | 0.2  (0.18-0.21) | 34 | 3.38  (2.34-4.72) | 17.3  (12.3-24.3) | 12 | 1.19  (0.62-2.08) | 6.1  (3,.4-10.8) |
| Malaria blood film positive | 7610 | 1.56  (1.52-1.59) | 68 | 6.75  (5.24-8.56) | 4.3  (3.4-5.5) | 30 | 3.0  (2.01-4.25) | 1.9  (1.3-2.7) |
| Severe anemia | 1470 | 0.3  (0.29-0.32) | 130 | 12.9  (10.8-15.3) | 42.9  (35.9-51.4) | 48 | 4.8  (3.51-6.32) | 15.9  (11.9-21.1) |
| Transfused | 1623 | 0.33  (0.32-0.35) | 115 | 11.4  (9.43-13.7) | 34.4  (28.5-41.6) | 50 | 5.0  (3.7-6.55) | 15.0  (11.3-19.8) |
| Died | 1089 | 0.22  (0.21-0.24) | 31 | 3.08  (2.09-4.37) | 13.8  (9.7-19.8) | 1 | 0.1  (0.003-0.55) | 0.4  (0.1-3.2) |

**Supplementary Figure.**

25619 pediatric admissions between 1^st^ January 2000 and 31^st^ December 2004

5019 lived outside the study area

20600 lived within the study area

26 older than 13 years

20574 aged 0-13 years

1710 no stored sample for sickle genotyping or genotyping unsuccessful

18873 cases included in this study
